# Expression Levels of a Kinesin-13 Microtubule Depolymerase Modulates the Effectiveness of Anti-Microtubule Agents

**DOI:** 10.1371/journal.pone.0011381

**Published:** 2010-06-30

**Authors:** Gregory V. Schimizzi, Joshua D. Currie, Stephen L. Rogers

**Affiliations:** 1 Department of Biology, The University of North Carolina at Chapel Hill, Chapel Hill, North Carolina, United States of America; 2 Carolina Center for Genome Sciences, The University of North Carolina at Chapel Hill, Chapel Hill, North Carolina, United States of America; 3 Lineberger Comprehensive Cancer Center, The University of North Carolina at Chapel Hill, Chapel Hill, North Carolina, United States of America; Roswell Park Cancer Institute, United States of America

## Abstract

**Background:**

Chemotheraputic drugs often target the microtubule cytoskeleton as a means to disrupt cancer cell mitosis and proliferation. Anti-microtubule drugs inhibit microtubule dynamics, thereby triggering apoptosis when dividing cells activate the mitotic checkpoint. Microtubule dynamics are regulated by microtubule-associated proteins (MAPs); however, we lack a comprehensive understanding about how anti-microtubule agents functionally interact with MAPs. In this report, we test the hypothesis that the cellular levels of microtubule depolymerases, in this case kinesin-13 s, modulate the effectiveness of the microtubule disrupting drug colchicine.

**Methodology/Principal Findings:**

We used a combination of RNA interference (RNAi), high-throughput microscopy, and time-lapse video microscopy in *Drosophila* S2 cells to identify a specific MAP, kinesin-like protein 10A (KLP10A), that contributes to the efficacy of the anti-microtubule drug colchicine. KLP10A is an essential microtubule depolymerase throughout the cell cycle. We find that depletion of KLP10A in S2 cells confers resistance to colchicine-induced microtubule depolymerization to a much greater extent than depletion of several other destabilizing MAPs. Using image-based assays, we determined that control cells retained 58% (±2%SEM) of microtubule polymer when after treatment with 2 µM colchicine for 1 hour, while cells depleted of KLP10A by RNAi retained 74% (±1%SEM). Likewise, overexpression of KLP10A-GFP results in increased susceptibility to microtubule depolymerization by colchicine.

**Conclusions/Significance:**

Our results demonstrate that the efficacy of microtubule destabilization by a pharmacological agent is dependent upon the cellular expression of a microtubule depolymerase. These findings suggest that expression levels of Kif2A, the human kinesin-13 family member, may be an attractive biomarker to assess the effectiveness of anti-microtubule chemotherapies. Knowledge of how MAP expression levels affect the action of anti-microtubule drugs may prove useful for evaluating possible modes of cancer treatment.

## Introduction

Microtubules (MTs) are cytoskeletal filaments composed of non-covalent polymers of αβ-tubulin heterodimers [1], [2]. During interphase, microtubules perform essential cellular processes including organization of the cytoplasm, definition of cellular shape and structure, positioning the nucleus and other cellular organelles, and serving as structural components of cilia and flagella [1]. MTs are 25 nm-wide hollow structures, composed of thirteen polarized, linear protofilaments formed by the head-to-tail association of αβ-tubulin heterodimers. The structural polarity of microtubules confers directionality to microtubule-associated motors such as kinesin and dynein, as well as for the localization of some microtubule-associated proteins (MAPs) [3].

While MT tracks form essential intracellular highways for the transport of cellular material, they are not static structures. MTs are highly dynamic and exhibit alternating phases of growth and shrinkage, a behavior known as dynamic instability [1]. This switching behavior between periods of growth and shrinkage is influenced by the binding and hydrolysis of guanosine triphosphate (GTP) by the β-subunit of αβ-tubulin heterodimers within microtubules. GTP-bound tubulin exhibits a straight conformation which favors incorporation into the growing plus end of the microtubule by allowing lateral interaction between tubulins of adjacent protofilaments. Once β-tubulin undergoes GTP hydrolysis shortly after incorporation into the microtubule lattice, the heterodimer adopts a bent conformation which precludes these lateral interactions and favors microtubule catastrophe [1]. The presence or absence of a stabilizing “cap” of GTP-tubulin at the plus ends of microtubules influences the dynamicity of individual polymers [4]. Dynamic instability is essential for a variety of processes during interphase, including cell polarization and subsequent movement, and the attachment of microtubules to various cellular targets for intracellular trafficking. The dynamic nature of the microtubule cytoskeleton is also of vital importance during mitosis as it allows cells to assemble a mitotic spindle and to find kinetochore attachment sites via a “search and capture” mechanism [1], [5].

In addition to the normal fluctuations in microtubule dynamics brought about by tubulin's intrinsic GTPase activity, dynamic instability can also be enhanced or suppressed by the actions of MAPs. MAPs may be categorized into two types: conventional stabilizing MAPs, which bind along the microtubule lattice and act to bundle microtubules, and MT plus end-interacting proteins (+TIPs), which preferentially associate with plus ends to regulate growth and shrinkage. Some of these +TIPs, such as members of the XMAP215/Dis1 family, act as MT polymerases that potently stimulate the growth of MTs. Other +TIPs, such as the kinesin-13 sub-family, act to trigger MT catastrophe and act as potent depolymerases. Thus, the MT plus end is a key site for the regulation of MT behavior by MAPs that exhibit antagonistic activities.

Drugs that actively disrupt MT dynamics prevent the proper formation of the mitotic spindle and in doing so trigger apoptosis in dividing cells. As transformed cells typically divide much more quickly than non-transformed cells, anti-microtubule agents are often useful as chemotherapeutic cancer treatments [4], [6]. Thus, small molecules that disrupt the mitotic spindle have been attractive targets to specifically kill cancer cells [7]–[9]. Many anti-MT agents, such as colchicine, nocadazole, and vinblastine, function by binding to αβ-tubulin heterodimers to interfere with MT polymerization and have proven to be highly effective at preventing cancer cell proliferation [10]–[12]. Despite the importance of MT-disrupting small molecules as chemotherapeutics, we know very little about whether these drugs act in concert with MAPs or how MAP expression levels might affect the efficacy of drug treatment.

Previous research suggested that variable expression levels of the mammalian kinesin-13, MCAK, altered the effectiveness of anti-MT drugs on human cancer cells [9]. In this study, we present the effects of depletion and of kinesin-13 family members and other MAPs in *Drosophila* S2 cells treated with anti-microtubule drugs. Knowledge of how MAPs such as these modulate anti-microtubule drug effectiveness could be used to assess possible modes of chemotherapy treatment for cancer patients. These experiments suggest that the kinesin-13, KLP10A, is of particular importance for the activity of the anti-MT drug colchicine. *Drosophila* S2 cells exhibit increased resistance to colchicine-induced MT depolymerization when depleted of this protein and increased susceptibility to drug treatment upon over-expression of KLP10A-GFP. Our results suggest that expression levels of Kif2A, the human kinesin-13 family member, may be a useful biomarker to predict the effectiveness of anti-microtubule agents in various cancers. Moreover, they predict that allosteric small molecule activators of kinesin-13 family members could act to potentiate the effectiveness of anti-microtubule drugs in chemotherapeutic strategies.

## Results

### RNAi-mediated depletion of KLP10A confers resistance to colchicine treatment

**Figure 1:**
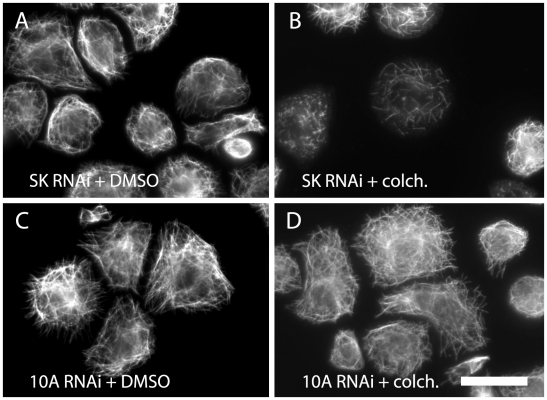
RNAi-mediated depletion of KLP10A protects microtubules from depolymerization by colchicine. (A,B) *Drosophila* S2 cells were cultured with negative control dsRNA for seven days, then either treated with DMSO (A) or 1 µM colchicine (B) for 1 hr and processed for immunofluorescence to visualize the microtubule cytoskeleton. Note the short microtubule fragments remaining after colchicine treatment in (B). (C,D) S2 cells were cultured with dsRNAs to deplete KLP10A for seven days, then treated with either DMSO (C) or colchicine (D) as described. Note that microtubules in (D) closely resemble those in control cells (C). Bar = 5 µm.

In order to determine if the expression level of MAPs affected the sensitivity of S2 cells to anti-MT treatment, we began our study by performing a targeted RNAi screen to deplete cells of known MT-destabilizing proteins. The proteins examined were the three *Drosophila* kinesin-13s (KLP10A, KLP59C, and KLP9D). This group was chosen due to the fact that they exhibit microtubule destabilizing functions in a variety of contexts and they were therefore considered as potential candidates to alter anti-microtubule drug action. Furthermore, previous work suggested that kinesin-13s may antagonize anti-microtubule drug action during mitosis, thus the *Drosophila* kinesin-13 family members were of particular interest [9]. Initial analysis of the affects of protein knockdowns on anti-microtubule drug action were conducted by visual examination of the morphology of the microtubule cytoskeleton in colchicine treated vs. DMSO treated cells of each RNAi condition. Control RNAi-treated (SK) cells exhibited fairly extensive microtubule loss upon 1 µM colchicine treatment for 1 hour (Fig. 1 A,B). KLP10A RNAi knockdowns, on the other hand, were relatively resistant to drug treatment, with microtubules that closely resembled DMSO control-treated KLP10A knockdowns (Fig. 1 C,D). S2 cells depleted of KLP10A appeared remarkably resistant to colchicine in this initial observation. For the other RNAi knockdowns, no dramatic changes in sensitivity to colchicine treatment were observed by eye. While this experiment provided convincing images suggesting the involvement of KLP10A in colchicine-induced microtubule depolymerization, we developed additional assays for more quantitative analyses of MT polymer levels.

### Quantitative analysis of colchicine resistance using high-throughput microscopy

**Figure 2:**
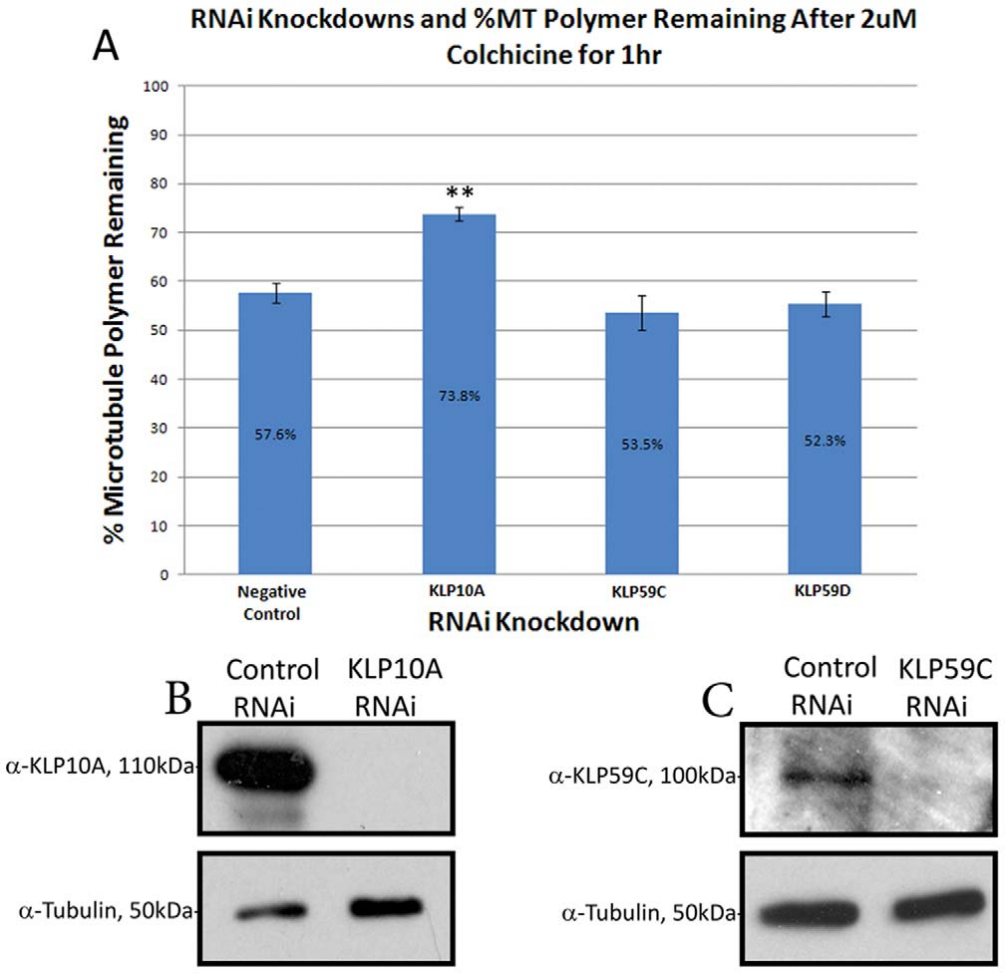
Total microtubule polymer levels are protected from colchicine upon depletion of KLP10A in cell populations as determined using high-throughput microscopy. (A) Populations of cells were either treated with SK dsRNA as a negative control, KLP10A dsRNA, KLP59C dsRNA, or KLP59D dsRNA for 7 days and processed for immunofluorescence to visualize microtubules and DNA in 96-well plates. Individual wells were scanned using a high-throughput microscope to acquire images of 1700-2000 cells per well. The imaging system software was programmed to identify individual nuclei in the DNA channel and to dilate nuclear objects to generate masks of individual cell outlines. These cellular masks were then used to measure the average fluorescence pixel intensity per cell in the microtubule channel for each treatment. The graph is a representative replicate of the results obtained for 3 independent duplicate experiments. When comparing %MT polymer remaining after drug treatment, only KLP10A knockdowns showed statistically significant differences from controls (p<0.0001). KLP59C and KLP59D knockdowns were not statistically significant from controls with respect to the amount of MT polymer remaining after colchicine treatment (p = 0.1942 and p = 0.3651, respectively). Western Blots of cell lysates are shown demonstrating KLP10A knockdown (B) and KLP59C knockdown (C) by RNAi. Using densitometry we calculated RNAi knockdown efficiencies to be 99% reduction of KLP10A and 76% reduction of KLP59C protein levels. A parallel tubulin blot is shown as a loading control.

After the initial qualitative observation of the resistance to colchicine in cells lacking KLP10A, we used high-throughput image cytometry to measure this effect quantitatively. Our strategy was to use RNAi to deplete kinesin-13 protein levels and prepare samples for immunofluorescence using fixation protocols that simultaneously extracted soluble tubulin so that only microtubule polymer contributed to the fluorescence signal from the samples. To measure the effect of KLP10A depletion on the efficacy of colchicine-induced microtubule depolymerization, cells were plated in 96-well format, treated with 2 µM colchicine, and stained for DNA and microtubules. We then used a high-throughput imaging system that autofocused on and identified cells by fluorescence in the DNA channel and then dilated an image mask of the nucleus in the MT channel to acquire a measurement of the average microtubule immunofluorescence pixel intensity across 1,700–2,000 cells in each well. Measurement of average microtubule immunofluorescence provided a quantitative measure of the relative sensitivity to colchicine treatment in cells depleted of the collection of MT destabilizing factors. S2 cells treated with negative control SK dsRNA retained 58% (±2%SEM) of total microtubule polymer after colchicine treatment as compared to control DMSO-treated cells. KLP10A RNAi knockdowns retained a much larger portion of total microtubule polymer after drug treatment: 73% (±1%SEM) (Fig. 2A). Depletion of the other kinesin-13s, KLP59C and KLP59D, did not result in statistically significant differences from controls with respect to cellular susceptibility to drug treatment (Fig. 2A,). The effectiveness of these RNAi knockdowns was evaluated by western blot analysis of cell lysates from each RNAi condition (Fig. 2B, 2C). KLP10A RNAi resulted in a 99% decrease in protein levels and KLP59C RNAi resulted in a 76% reduction in protein levels. We were unable to validate KLP59D knockdown by western blot analysis as we lacked specific antibodies to the protein and have not yet generated KLP59D-GFP constructs which could be used for western blotting. However, previous research suggests that depletion KLP59D does not produce an interphase phenotype [13], supporting the idea that KLP59D would have no effect on colchine-induced MT depolymerization during interphase. From these data, we conclude that depletion of KLP10A protects cells from MT destabilization by colchicine.

### Over-expression of KLP10A in S2 cells results in greater sensitivity to colchicine treatment

**Figure 3:**
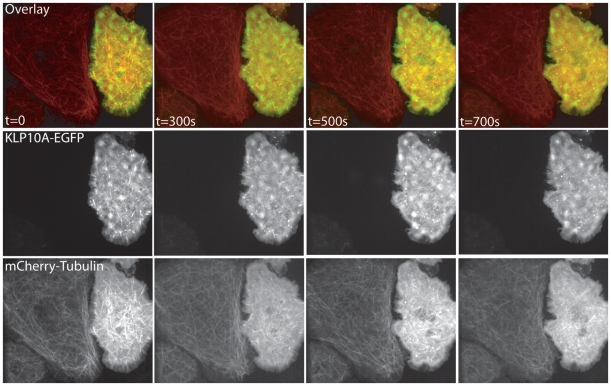
Increased KLP10A expression levels confers sensitivity to colchicine as determined using live-cell microscopy. S2 cells were cotransfected with plasmids encoding KLP10A-EGFP and mCherry-tubulin and image using time-lapse spinning disk confocal microscopy. Images were acquired at 10 second intervals and cells were perfused with 4 µM colchicine approximately 170s in to the sequence. Note that the rate of microtubule depolymerization is greater in cells with the highest levels of expression of KLP10A-EGFP. By approx. t = 500 s the cell expressing high levels of KLP10A-EGFP has nearly no MT polymer while the MT cytoskeleton in the neighboring cell remains relatively unaffected.

As the depletion of KLP10A conferred increased resistance to colchicine treatment in S2 cells, we next examined the susceptibility of MTs to colchicine in S2 cells over-expressing KLP10A. We co-transfected S2 cells with expression vectors for KLP10A-EGFP and mCherry-tubulin driven by the metallothionine promoter and induced expression overnight with 100 µM copper sulfate. Transient transfection typically produces a population of cells with a range of expression levels of the constructs due to variation in the number of copies of the plasmid delivered. We then performed time-lapse imaging on a spinning disk confocal microscope and observed MT depolymerization during perfusion with colchicine. S2 cells expressing high levels of KLP10A-GFP, as determined by relative GFP fluorescence intensities, exhibited a much greater sensitivity to colchicine treatment, as their MT polymer levels decreased at a faster rate than MTs in cells not expressing the KLP10A transgene (Fig. 3, Movie S1). These time-lapse movies suggest that over-expression of KLP10A-GFP causes an increase in the kinetics and extent of microtubule depolymerization by colchicine.

**Figure 4:**
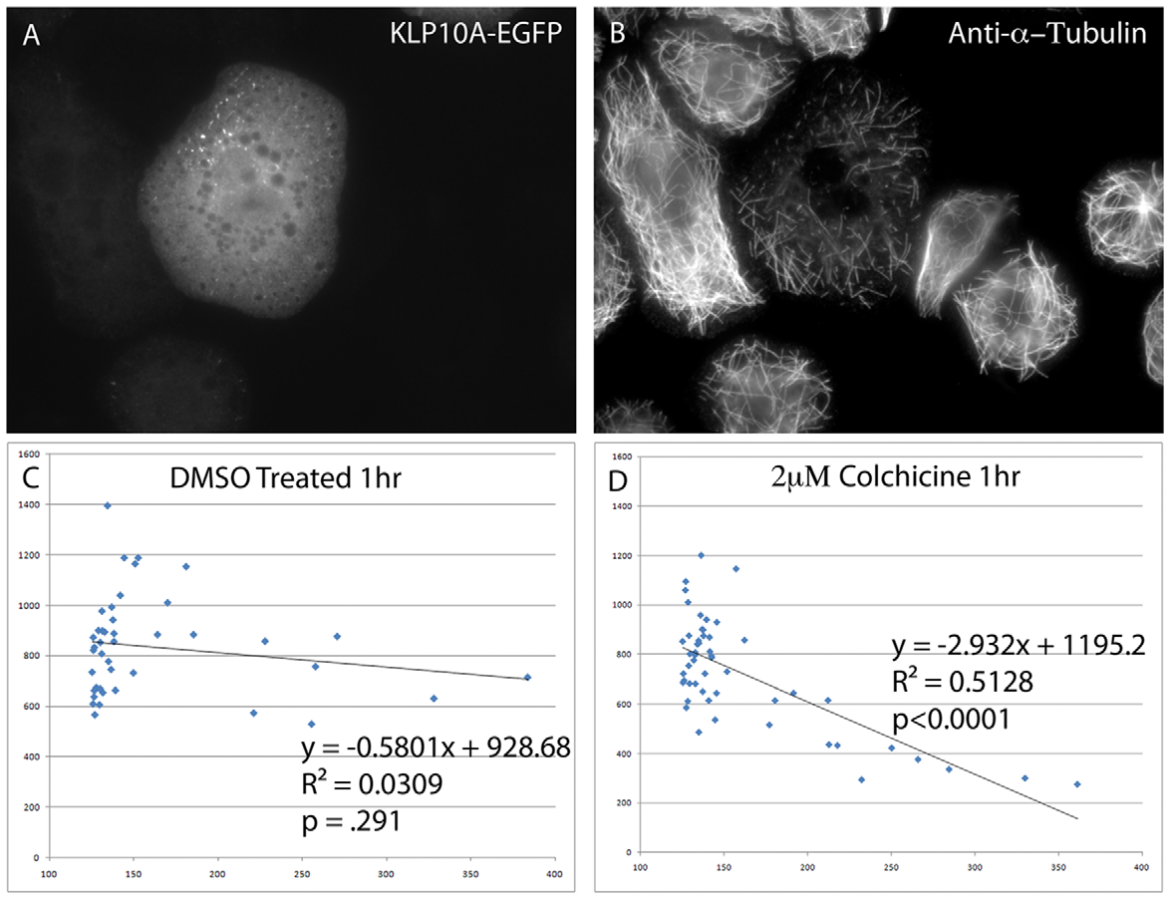
Overexpression of KLP10A-GFP in *Drosophila* S2 cells confers increased susceptibility to colchicine treatment. S2 cells were transfected, induced to express KLP10A-GFP, treated with 1 µM colchicine for 1 hour, then processed for immunofluorescence to visualize microtubules. A representative transfected cell is shown in the tubulin channel (A) and GFP channel (B). Note extensive loss of microtubules in (A). In order to test for a correlation between expression levels of KLP10A-GFP and the extent of microtubule depolymerization, individual transfected cells were imaged in both channels and microtubule polymer levels and KLP10A-GFP expression levels were determined by measuring average fluorescent pixel intensities per cell. Bar  = 5 µm. The results are plotted for control DMSO-treated cells (C) and colchicine-treated cells (D). The amount of KLP10A-GFP in DMSO treated cells (C) did not show a statistically significant effect on the amount of MT polymer present (p = 0.291). However, when transfected cells were treated with colchicine (D) a strong negative correlation is observed between the amount of KLP10A-GFP present and the amount of MT polymer present (p<0.0001).

**Figure 5:**
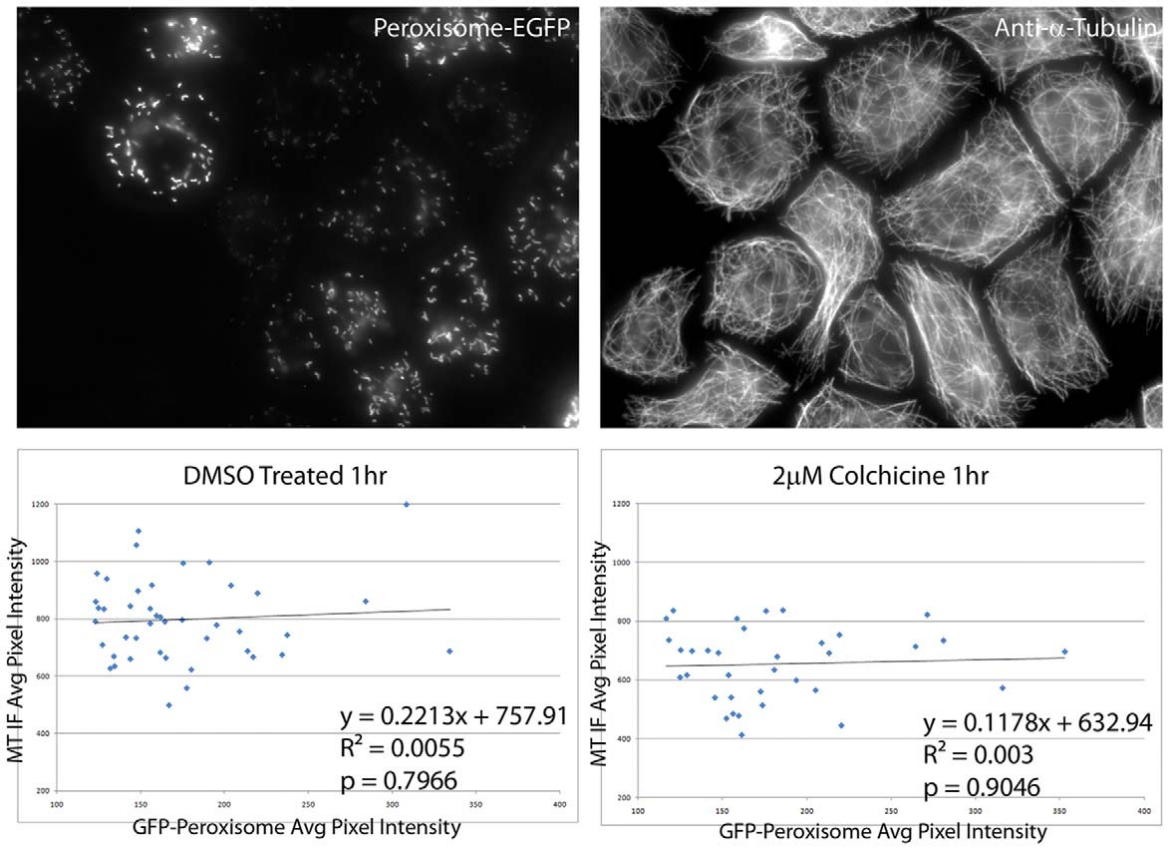
GFP expression alone does not protect microtubules from colchicine. As a negative control, S2 cells were transfected with a plasmid expressing GFP targeted to peroxisomes, either treated with DMSO or colchicine, and processed as described in Figure 3 to visualize microtubules (A) and peroxisomes (B). Bar  = 5 µm. The plots show average microtubule pixel intensity per cell plotted against GFP peroxisome average pixel intensity per cell. The amount of Peroxisome-GFP did not show a statistically significant effect on the amount of MT polymer present in DMSO-treated cells (p = 0.7966, C) or colchicine-treated cells (p = 0.9046, D).

Time-lapse imaging provided compelling support for the involvement of KLP10A in colchicine-induced microtubule depolymerization. In order to correlate the relationship between KLP10A expression levels and susceptibility to drug treatment, S2 cells were transfected with only KLP10A-GFP and induced to express high levels of the construct. We then treated the cells with colchicine, fixed them, and stained for microtubules. We acquired images of cells expressing a range of KLP10A-GFP and measured the average pixel intensities for microtubule immunofluorescence and KLP10A-GFP fluorescence (Fig. 4 A,B). We found a very clear correlation between the level of expression of KLP10A-GFP and increased sensitivity to colchicine in S2 cells (m = −2.932, p<0.0001, Fig. 4D). We treated parallel samples with DMSO as a negative control for colchicine treatment and found only a slight negative correlation between the amount of KLP10A-GFP and the amount of microtubule polymer in each cell (m = −0.5801, p = .291, Fig. 4C), however, this was to be expected as KLP10A is known to function as a microtubule de-stabilizer [14]. We also controlled for the possibility that expression of GFP itself rendered cells more susceptible to colchicine by transfecting S2 cells with a GFP-peroxisome marker and subjected to the same procedure (Fig. 5). As shown in Figure 5, the presence of GFP in cells had no correlative effect on colchicine-induced microtubule depolymerization. This also demonstrates the amount of colchicine-induced MT depolymerization in cells without the presence of exogenous KLP10A. Colchicine-induced MT depolymerization appears less extensive in cells expressing only endogenous levels of KLP10A.

## Discussion

The data presented here demonstrate that the expression level of a particular MT depolymerase alters the effectiveness of the anti-MT drug colchicine. RNAi depletion of KLP10A in S2 cells confers greater resistance to colchicine-induced MT depolymerization, and likewise, over-expression of a KLP10A construct causes increased cellular susceptibility to colchicine treatment. While some resistance to drug-induced microtubule depolymerization might be expected when cells are depleted of a protein that promotes microtubule depolymerization, neither of the other kinesin-13 family members tested caused such a drastic alteration of cellular susceptibility to drug treatment. This suggests that KLP10A may have a unique role in this process. This supports the idea that Kif2A, the human homologue of KLP10A, may be an attractive biomarker for assessing the effectiveness of potential anti-MT chemotherapy treatments. In addition, the development of drugs or small molecules that alter kinesin-13 activity could prove therapeutically useful in combination with anti-MT drugs. However, further investigation into this matter is still needed to determine the effects of altered expression of other kinesin-13s on colchicine action, and how depletion and over-expression of these proteins affects the action of other anti-MT drugs.

A potential mechanism that would explain the results presented in this paper has become apparent. Previous research has shown that kinesin-13s bind preferentially to GDP-tubulin in curved protofilaments at the plus ends of MTs [15], [16]. Colchicine is known to bind at the intra-dimer cleft in between the α- and β-subunits of tubulin heterodimers and induce a conformational change in tubulin from the straight GTP-bound conformation to one that mimics the bent GDP-bound conformation. These soluble tubulin-colchicine complexes are then incorporated into microtubule polymers and promote microtubule catastrophe [10]. An increased presence of tubulin-colchicine complexes in microtubule ends that mimic the natural substrate of KLP10A may provide KLP10A with a higher cellular concentration of potential substrate on which to act. KLP10A may then be able to localize more efficiently to these microtubules with large amounts of tubulin-colchicine complexes and cause rapid microtubule depolymerization. When KLP10A is not present, however, the cellular machinery appears to be less capable of depolymerizing microtubules. This suggests that KLP10A is either recruited more extensively to these positions, or is more active during colchicine-induced MT depolymerization, than other kinesin-13s. These details remain to be determined.

In the future, we will continue to investigate the effects of altered expression levels of MAPs on the susceptibility of S2 cells to other anti-MT drugs. Specific drugs of interest include the vinca-alkaloid, vinblastine, and taxol. Furthermore, the effect of overexpression of KLP59C and KLP59D on the susceptibility of S2 cells to colchicine remains to be tested. As cellular depletion of these proteins did not cause a drastic change in susceptibility to colchicine treatment, we predict that overexpression will similarly have little effect on the action of colchicine. It will also be interesting to determine if the presence of stabilizing MAPs required for MT growth also confer resistance to anti-MT drugs. Experiments testing these ideas are currently underway in the laboratory.

Colchicine is not currently used to treat cancer, but its potential for use as a chemotherapy drug has received significant investigation. However, many of the anti-microtubule drugs that are used as chemotherapy treatments cause nearly identical cellular phenotypes at similar concentrations and have been shown to bind both tubulin heterodimers and the microtubule lattice in a similar manner to colchicine [10]. This suggests that other anti-microtubule drugs, such as the vinca alkaloids, may also exhibit variation in effectiveness when expression levels of specific MAPs are altered.

Many anti-microtubule drugs are currently used as chemotherapy treatments and broader knowledge as to how the expression levels of MAPs affect the activity of these drugs would be especially valuable. If the gene expression profiles of proteins that effect anti-microtubule drug action can be obtained for different cancer sub-types, more appropriate and effective modes of chemotherapy treatment could be selected for cancer patients. Drugs could potentially be prescribed that would optimize treatment outcomes and minimize side-effects.

## Materials and Methods

### S2 Cell Culture and Double Stranded RNAi

*Drosophila* S2 cells were obtained from the Drosophila Genome Resource Center (Bloomington, IN). S2 culture and RNAi were performed as previously described [17].

We used the following primer sets to perform RNAi. A DNA sequence from the pBluescript SK plasmid (SK) that has no homology in the *Drosophila* genome was used as a negative control for RNAi. RNAi against the mitotic kinesin, Pavarotti, was performed in parallel as a positive control as they fail to undergo cytokinesis and become multinucleated, providing a visual assay of knockdown effectiveness. For RNAi, 7 µg of dsRNA/mL media was added to cultured S2 cells once/day for 7–9 days. Templates for *in vitro* transcription (Promega T7 RiboMax kit) were generated by using primers that began with the T7 promoter on the 5′ primer 5′-TAATACGACTCACTATAGGG-3′ and continue with gene specific sequences listed here. The primers were (forward and reverse): Pavarotti (5′ – AATGTGTTCTGTCGAGTGCG – 3′ and 5′ – AGCAAAACGCTGGTTCATCT – 3′), SK (5′-TAAATTGTAAGCGTTAATATTTTG-3′ and 5′-AATTCGATATCAAGCTTATCGAT-3′), KLP10A: (5′-ATGATTACGGTGGGGCA-3′ and 5′-GACATCGATCT CCTTGCG-3′), KLP59C: (5′-ATGGATAAGTTGTCGATCG-3′ and 5′-ACCAGGTTCACATGCTTGCG-3′), KLP59D: (5′-GGATCGCATCAAAATTGG-3′ and 5′-CGTAGACCAGCGCATTG-3′).

### Immunofluorescence Microscopy

RNAi-treated S2 cells were plated on Concanavalin A (ConA) coated coverslips and fixed in 10% formaldehyde in BRB80 buffer (80 mM PIPES, 1 mM magnesium chloride, 1 mM EDTA, pH 6.9) at room temperature for 10 min. For high throughput analysis, cells were fixed in 10% formaldehyde, 0.1% Saponin in BRB80 buffer for 3 min, and then in 10% formaldehyde in BRB80 (without Saponin) for 7 min. The presence of this additional step with detergent for the first three minutes (0.1% Saponin) further removed the cytosolic pool of unpolymerized tubulin that would otherwise contribute to background fluorescence when imaging and quantifying MT pixel intensities. The cells were then washed in phosphate buffered saline (PBS) plus 0.1% Triton X100 (PBST), followed by blocking with 5% normal goat serum (NGS) in PBST. The primary antibody used was an anti-α-tubulin antibody (DM1α, Sigma-Aldrich, St. Louis, MO). After treatment with fluorescently labeled secondary antibodies (Cy3 – Jackson ImmunoResearch Laboratories) and AlexFluor488 Phalloidin (Invitrogen) for 1 hr, cells were stained for DNA with Hoechst (Invitrogen) at a final concentration of 10 µg/mL. Samples were mounted in Dako Fluorescent Mounting Medium and imaged using a Nikon Eclipse Ti-E microscope equipped with a 100× 1.45NA Nikon objective and CoolSnap HQ cooled CCD camera.

### High-Throughput Microscopy for Quantification of Microtubule Polymer Levels in RNAi Knockdown Cells

RNAi knockdown S2 cells were seeded on ConA coated 96-well dishes (Nunc-Clear-96 plates) at a density of 30,000 cells per well for ∼1.5 hr, treated with 2 uM colchicine in Schneider's Medium for 1 hr, stained (as described in Immunofluorescence Microscopy), and scanned with an Array Scan V (Cellomics) equipped with a 40× NA 0.95 objective and a cooled charge-coupled device camera (ORCA-ER; Hamamatsu Photonics). Images of 1,700–2,000 cells per well were acquired and analyzed using vHCS View (Cellomics). Objects in each well were identified by DNA staining and average object microtubule fluorescence pixel intensity measurements for each well were determined from these images.

### S2 Cell Transfections and Live Imaging

Fluorescent constructs of mCherry-Tubulin, KLP10A-GFP, and a GFP Peroxisome marker in Invitrogen pMT vectors were transfected into *Drosophila* S2 cells via electroporation using the Nucleofector II (Amaxa) apparatus. Cells were allowed ∼24 hrs in Schneider's medium to recover after transfection. Expression of the constructs was then induced for ∼18 hrs by addition of 100 µM CuSO_4_.

Induced cells were plated on ConA coated MatTek glass-bottom dishes for at least 1 hr. before observation. Samples were imaged using a 100X NA 1.45 PlanApochromat objective (Nikon) using a spinning disc confocal (Yokogawa: Perkins-Elmer) mounted on a microscope (Eclipse TE300: Nikon) with a z-focus motor (Prior Scientific), and excitation and emission wheel controlled by a Lambda 10-2 controller (Sutter Instrument Co.), an interline transfer-cooled charge-coupled device camera (ORCA-ER, Hamamatsu Photonics), and emission filters (Semrock). Images were acquired for time-lapse movies once every 10 s for approx. 15 min time spans using MetaMorph software (MDS Analytical Technologies). Colchicine was added to the medium to a final concentration of 4 µM at about frame 17 (approx. 170 s) and microtubule depolymerization was recorded in time-lapse videos.

## Supporting Information

Movie S1 S2 cells were cotransfected with plasmids encoding KLP10A-EGFP and mCherry-tubulin and image using time-lapse spinning disk confocal microscopy. Images were acquired at 10 second intervals and cells were perfused with 4 µM colchicine approximately 170s in to the sequence. Note that the rate of microtubule depolymerization is greater and more extensive in the cell expressing high levels of KLP10A-EGFP.
(5.66 MB MOV)
